# Plasminogen activator inhibitor-1: a risk factor for deep vein thrombosis after total hip arthroplasty

**DOI:** 10.1186/s13018-018-0716-2

**Published:** 2018-01-10

**Authors:** Ju Tang, Wei Zhu, Xiaoliang Mei, Zhenxiang Zhang

**Affiliations:** Department of Joint Surgery, Taizhou People’s Hospital, Medical School of Nantong University, No. 210 Yingchun Road, Taizhou City, Jiangsu Province, 225300 China

**Keywords:** Total hip arthroplasty, Deep vein thrombosis, Plasminogen activator inhibitor-1, Risk factor

## Abstract

**Background:**

The onset of deep vein thrombosis (DVT) in patients after total hip arthroplasty (THA) may expand or enlarge and subsequently lead to significant mortality. The objective of this study was to investigate potential risk factors for DVT in patients after THA.

**Methods:**

Eligible patients with hip joint diseases who were scheduled for unilateral primary THA at our hospital were prospectively included into this study. The demographic and clinical features, preoperative plasma biomarkers were detailed, recorded, and compared. The multiple logistic regression analysis was used to evaluate potential risk factors for DVT.

**Results:**

A total of 214 subjects were enrolled into our study cohort for the final analysis, and 23 of them have suffered DVT with an incidence of 9.5%. The performance of logistic regression analysis showed that preoperative expression of plasminogen activator inhibitor-1 (PAI-1) was an independent risk factor for the onset of DVT in patients after THA (OR 1.18, 95% CI 1.04–1.29; *p* = 0.011).

**Conclusions:**

Our study indicated preoperative plasma PAI-1 expression as an independent risk factor for DVT in patients who underwent THA.

## Background

Venous thromboembolism (VTE) is recognized as one of most common complications during patients undergoing total hip arthroplasty (THA) and can be closely related to postoperative mortality with uncertain prophylaxis [1]. Previous studies in western populations have reported the incidence of deep vein thrombosis (DVT) in THA patients can be as high as 40–60% with no appropriate chemoprophylaxis [2]. Another study in Asian countries has reported the incidence of DVT around 8%, which is significantly lower than western countries [3]. Accurate, rapid diagnosis and valid prediction of DVT during the perioperative period are essential due to its significant impact on morbidity and mortality [4]. Previous studies have established D-dimer as an effective biomarker for DVT [5, 6], however, another study conducted by Thomas et al. did not support the predictive value of D-dimer in symptomatic DVT after total joint arthroplasty [7]. The occurrence of thrombosis can be ascribed to the imbalance between coagulation and fibrinolytic factors [8]. Previous studies have revealed that plasminogen activator inhibitor-1 (PAI-1), a central regulative factor for the fibrinolytic system, has been considered as a critical role in hemostatic clot stabilization and vascular thrombosis determination [9, 10]. Impaired fibrinolytic function induced by elevated PAI-1 expression is commonly observed in patients with thrombotic disease. Previous reports by Wiman B have indicated preoperatively increased PAI-1 concentration as a potential predicator for postoperative DVT in subjects undergoing hip surgery with poorly understood pathophysiology [11]. Another study conducted in Indian patients has also shown a close association between raised PAI-1 level which is regulated by PAI-1 gene 4G/5G polymorphism and DVT through a hypoactive fibrinolytic pathway [12]. Reports by Joe et al. have showed the critical role of plasmin in thrombus resolution and the lack of PAI-1 closely correlates with greater vein wall fibrosis [13]. However, whether PAI-1 can serve as a risk factor for DVT in patients undergoing THA still remains unknown. The purpose of our present study was to investigate potential risk factors for DVT after THA.

## Methods

### Patients

This prospective study protocol was approved by the Medical Institutional Ethics Committee of Taizhou People’s Hospital and Jiangsu province. Eligible patients who underwent unilateral primary THA at Taizhou People’s Hospital from May 2012 to January 2017 were prospectively enrolled. The written informed consent was obtained from each participant. The exclusion criteria were described as follows: (1) with thromboembolism history; (2) ongoing antiplatelet or anticoagulation treatment for cerebrovascular or cardiac diseases; (3) with revision hip arthroplasty; (4) diagnosed with DVT before the surgery with Doppler ultrasonography. Antiplatelet or anticoagulant medications were discontinued for 1 week prior to surgery.

Antiplatelet or anticoagulant medications (such as low molecular heparin) were also not routinely conducted postoperatively. Elastic stockings wearing and early lower limb function exercise were the only postoperative antithrombotic prophylactic treatments for the enrolled patients until the onset of DVT by Doppler ultrasonography or clinical symptoms.

The demographic characteristics (age, sex, etc.) and preoperative comorbidities (diabetes mellitus, anemia, etc.) from all enrolled patients were noted in details at admission. The operation-related indexes including operation side, American Society of Anesthesiologists (ASA) physical status, etc. were also recorded. DVT in distal and proximal veins was evaluated in each participant on preoperative, postoperative day 7 and day 30 with Doppler ultrasonography. The time from operation to DVT was also noted. The definition of DVT was confirmed according to previous studies [14], “new diagnosis of thrombus or blood clot in the superficial or deep venous system coupled with inflammation or not.”

### Blood sampling and laboratory examinations

The preoperative (1 day before the surgery) blood samples were collected from each enrolled patient into tubes containing ethylenediaminetetraacetic acid (EDTA). Then plasma samples were isolated and stored at − 80 °C for use. The plasma D-dimer, tissue factor (TF), microparticle-tissue factor (MP-TF), and thrombin-activatable fibrinolysis inhibitor (TAFI) (Abcam Co., Cambridge, UK) levels were analyzed by enzyme-linked immuno sorbent assay (ELISA). Double antibody enzyme-linked immunosorbent assays were utilized for the measurement of plasma PAI-1 (Abcam Co., Cambridge, UK) concentrations. Plasma expressions of D-dimer were detected by latex agglutination turbidimetry using FIA8000 (Jidan Co., Nanjing, China) with the normal range of < 0.5 μg/mL. The preoperative hemocyte analyses including white blood cell (WBC) counts, hemoglobin, hematocrit, and albumin were also conducted. We also measured coagulation function indexes including platelet counts, fibrinogen, prothrombin time (PT), activated partial thromboplastin time (APTT), and prothrombin time–international normalized ratio (PT-INR).

### Statistical analysis

SPSS 19.0 software (SPSS Inc., Chicago, IL, USA) and GraphPad prism 5.0 (GraphPad Inc., San Diego, CA, USA). Data were expressed as mean (standard error) or number (*n*, %), respectively. Chi-square test or Fisher’s exact test was used for the comparison of qualitative data. Mann-Whitney *U* test or Student’s *t* test was utilized for quantitative data analysis appropriately. Multiple logistic regression analysis was used for the determination of potential risk factors contributed to the presence of DVT. A *p* value < 0.05 with bilateral probability was considered statistically significant.

## Results

### Demographic and clinical features

Of the 241 enrolled participants, 27 were excluded from this study: 4 declined to cooperate, 7 information missed, and 16 were ineligible due to the exclusion criteria. The remaining 214 subjects were enrolled into our study cohort for the final analysis, and 23 of them have suffered DVT with an incidence of 9.5% (5 patients were actually symptomatic). The detailed demographic and descriptive data of this study cohort were exhibited in Table 1. Sixteen of 23 patients with DVT were found early in the postoperative course (day 7), and no signs for pulmonary embolism in our series were observed. Those patients who suffered from DVT had a higher age than those without DVT (66.7 vs 62.6 years, *p* = 0.018). Differences were found between patients with or without DVT regarding preoperative comorbidities of diabetes mellitus, atrial fibrillation, and coronary artery disease. No statistical difference was found in the comparison of gender, BMI, ASA physical status, operation side, estimated blood loss, infusion volume, and urine volume between these two groups.

**Table 1:** Characteristics of patients with or without DVT after THA

Parameters	DVT		*p* value
	Yes	No	
Number (*n*)	23	191	–
Age (years)	66.7 ± 8.6	62.6 ± 7.7	0.018*
Sex (*n*, %)			
Male	8(34.8%)	70(36.6%)	
Female	15(65.2%)	121(63.4%)	0.861
BMI (kg/m^2^)	23.7 ± 3.1	23.5 ± 3.3	0.783
Comorbidities			
Diabetes mellitus	7(30.4%)	26(13.6%)	0.035*
Anemia	3(13.0%)	20(10.5%)	0.565
Atrial fibrillation	5(21.7%)	12(6.3%)	0.024*
Hypertension	8(34.8%)	55(28.8%)	0.552
Hyperlipidemia	3(13.0%)	20(10.5%)	0.721
Malignancy	2(8.7%)	11(5.8%)	0.636
Peripheral artery disease	1(4.3%)	6(3.1%)	0.554
Cerebrovascular disease	2(8.7%)	17(8.9%)	1.000
Coronary artery disease	5(21.7%)	15(7.9%)	0.047*
ASA physical status			
I–II	15(65.2%)	102(53.4%)	
III–IV	8(34.8%)	89(46.6%)	0.282
Operation side			
Left	10(43.5%)	109(57.1%)	
Right	13(56.5%)	82(42.9%)	0.215
Duration of surgery (min)	164.7 ± 31.3	150.8 ± 30.1	0.038*
Estimated blood loss (ml)	225.7 ± 167.4	217.4 ± 145.8	0.800
Infusion volume (mL)	2188.7 ± 897.5	2297.1 ± 974.2	0.612
Urine volume (mL)	894.7 ± 427.5	788.5 ± 511.4	0.340

*DVT* deep vein thrombosis, *THA* total hip arthroplasty, *ASA* American Society of Anesthesiologists, *BMI* body mass index. *p* values were calculated by chi-square test, Fisher’s exact test, Student’s *t* test, or Mann-Whitney *U* test

*p value < 0.05

### Preoperative biomarkers and DVT

The impact of preoperative biomarkers including hemocyte routine analysis, coagulation function indexes, plasma expressions of D-dimer, TF, MP-TF, TAFI, and PAI-1 on thrombogenesis was assessed. As shown in Table 2, the significant correlation with DVT was only observed between plasma concentrations of albumin, D-dimer, PAI-1, and TAFI. However, when comparing patients with and without DVT, no significant difference was identified between DVT and WBC count, hemoglobin, hematocrit, platelet count, fibrinogen, PT, APTT, PT-INR, creatinine, TF, or MP-TF.

**Table 2:** Preoperative biomarkers of patients with or without DVT after THA

Biomarkers	DVT		*p* value
	Yes	No	
Number (*n*)	23	191	–
WBC count (× 10 [9]/L)	6.2 ± 1.3	5.9 ± 1.6	0.388
Hemoglobin (g/L)	129.4 ± 16.5	132.2 ± 20.1	0.522
Hematocrit (%)	40.1 ± 4.1	40.8 ± 4.7	0.495
Platelet count (× 10 [9]/L)	221.1 ± 48.5	233.4 ± 64.1	0.375
Fibrinogen (mg/dl)	354.5 ± 137.7	338.7 ± 124.5	0.570
PT(s)	12.6 ± 1.9	13.1 ± 2.2	0.298
APTT(s)	30.4 ± 3.5	30.1 ± 4.4	0.753
PT-INR	0.98 ± 0.06	0.99 ± 0.08	0.563
Albumin (g/L)	37.1 ± 5.1	39.3 ± 4.8	0.040*
Creatinine (μmol/L)	69.5 ± 25.1	61.1 ± 20.7	0.074
D-dimer (μg/mL)	2.5 ± 1.9	1.7 ± 1.2	0.005*
TF (pg/mL)	24.4 ± 12.4	28.3 ± 14.4	0.215
MP-TF (pg/mL)	0.51 ± 0.19	0.46 ± 0.23	0.318
PAI-1(ng/mL)	43.1 ± 18.7	32.1 ± 12.5	< 0.001*
TAFI (%)	133.5 ± 30.1	147.4 ± 28.7	0.030*

*DVT* deep vein thrombosis, *THA* total hip arthroplasty, *WBC* white blood cell, *PT* prothrombin time, *APTT* activated partial thromboplastin time, *PT-INR* prothrombin time–international normalized ratio, *TF* tissue factor, *MP-TF* microparticle-tissue factor, *PAI-1* plasminogen activator inhibitor-1, *TAFI* thrombin-activatable fibrinolysis inhibitor. *p* values were calculated by Student’s *t* test or Mann-Whitney *U* test

*p value < 0.05

### Risk factors for DVT

All these nine risk factors mentioned above (Tables 1 and 2) were included into the multivariate logistic regression analysis. As shown in Table 3, the performance of logistic regression analysis showed that preoperative expression of PAI-1 was an independent risk factor for the onset of DVT in patients after THA (OR 1.18, 95% CI 1.04–1.29; *p* = 0.011).

**Table 3:** Multiple logistic regression analysis for DVT in patients after THA

Risk factors	Multiple logistic regression		
	OR	95% CI	*p* value
Age (years)	5.12	0.54–10.32	0.241
Diabetes mellitus	0.94	0.89–1.16	0.413
Atrial fibrillation	1.08	0.84–1.32	0.368
Coronary artery disease	0.91	0.71–1.25	0.587
Duration of surgery	1.22	0.61–2.21	0.612
Albumin	1.57	0.97–2.51	0.063
D-dimer	1.02	0.62–1.59	0.235
PAI-1	1.18	1.04–1.29	0.011*
TAFI	1.41	0.81–2.47	0.207

*DVT* deep vein thrombosis, *THA* total hip arthroplasty, *PAI-1* plasminogen activator inhibitor-1, *TAFI* thrombin-activatable fibrinolysis inhibitor, *CI* confidence interval, *OR* odds ratio

*p value < 0.05

## Discussion

The onset of DVT in patients after THA may expand or enlarge, and subsequently lead to pulmonary thromboembolism, a critical postoperative complication related to significant mortality [15]. Therefore, early diagnosis and predication of DVT is of great importance in patients undergoing THA, especially taking the limitations of current diagnostic tools into consideration. Although previous literature has established the incidence and risk factors of DVT after primary THA, a lack of consensus has been conducted [16]. Our results have indicated an incidence of 9.5% for DVT after unilateral primary THA, which was relatively higher than previous reports [3, 17]. We considered that the different races, ages, and inclusion criteria might lead to the different results. Moreover, the reported DVT prevalence might be overwhelmingly under-reported without postoperative routine ultrasound screening. The most significant finding of our present study was that PAI-1 was an independent risk factor for postoperative DVT after THA instead of D-dimer. Several previous studies have reported the predicative value of D-dimer for DVT after THA [18], which was not quite in accordance with our results. Moreover, a study containing 295 consecutive medical patients has also showed some routine blood tests (C-reactive protein, erythrocyte sedimentation rate, D-dimer, etc.) as risk factors for DVT [19]. Many existed confounding factors (advanced age, preoperative comorbidities, anesthesia projects, etc.) could be significantly associated with the conclusions [20, 21]. We considered that the different races, age ranges, cohort characteristics, preoperative comorbidities, inclusion criteria, and antithrombotic prophylactic treatments around the surgery could all be potential explanations for our different conclusions with some other reports.

As reported by previous literature, urokinase plasminogen activator (uPA) and tissue-type plasminogen activator (tPA) play critical actions in the primary mediation of intravascular fibrinolysis [22]. PAI-1 can modulate the plasminogen activation system by forming irreversible inhibitory complexes combining with uPA and tPA [23]. Previous studies have indicated the critical role of PAI-1 in hemostatic clot stabilization due to the close association between PAI-1 deficiency and abnormal bleeding [24]. Furthermore, elevated plasma expression of PAI-1 has been revealed as a significant risk factor for myocardial infarction [25] and DVT [26] by inhibiting endogenous fibrinolysis, which is consistent with our results. However, studies about correlations between PAI-1 concentrations and the maintenance of occlusive thrombosis still remain controversial. A study conducted in murine has reported no obvious correlation observed between time to thrombosis and PAI-1 deficiency [27]. Another study in mice of pulmonary embolism model has established that accelerated lysis of thrombus was associated with PAI-1 deficiency [28]. Important role of PAI-1 and vitronectin was also indicated in the vascular injury-associated occlusive thrombosis by regulating endogenous fibrinolysis [8]. Results from a large population-based prospective study by Folsom AR et al. failed to show a positive association between PAI-1 expression and risk of venous thrombosis [29]. In contrast, several other studies have offered evidence that postoperative thrombosis was associated with increased plasma PAI-1 expressions [30], which was in agreement with our results. PAI-1, derived from endothelial or platelet, is normally complexed to vitronectin and serves as an efficient inhibitor of thrombin, and activated protein *C. pai*-1 can modulate the activity of fibrinolytic system by competing with thrombomodulin and in combination with activated protein C inhibition [31].

## Conclusion

In conclusion, our study indicated preoperative plasma PAI-1 expression as an independent risk factor for DVT in patients after THA. More careful evaluation and aggressive anticoagulation were recommended to prevent postoperative DVT for those patients with elevated PAI-1 levels. In addition, further multi-center and large-scale studies are still required to verify the effectiveness of risk stratification for DVT following THA.

## Abbreviations

APTT: Activated partial thromboplastin time
ASA: American Society of Anesthesiologists
DVT: Deep vein thrombosis
EDTA: Ethylenediaminetetraacetic acid
ELISA: Enzyme-linked immuno sorbent assay
MP-TF: Microparticle-tissue factor
PT: Prothrombin time
PT-INR: Prothrombin time–international normalized ratio
TAFI: Thrombin-activatable fibrinolysis inhibitor
TF: Tissue factor
THA: Total hip arthroplasty
VTE: Venous thromboembolism
